# The low level of glucagon-like peptide-1 (glp-1) is a risk factor of type 2 diabetes mellitus

**DOI:** 10.1186/1756-0500-7-849

**Published:** 2014-11-26

**Authors:** Agus Lastya, Made Ratna Saraswati, Ketut Suastika

**Affiliations:** Division of Endocrinology and Metabolism, Department of Internal Medicine, Faculty of Medicine, Udayana University-Sanglah Hospital, Kamboja Street, Dangin Puri Kangin, No 8, 80233 Denpasar, Bali Indonesia

**Keywords:** Levels of GLP-1, Type 2 diabetes mellitus

## Abstract

**Background:**

Glucagon like peptide-1 (GLP-1), an incretin hormone, regulates glucose metabolism by inducing insulin secretion and suppressing glucagon secretion. The aim of the study is to assess the levels of fasting and post-prandial GLP-1 and their risk for T2DM. A case control study was conducted at the diabetes clinic Sanglah Hospital Denpasar Bali, involving 40 subjects who were native Indonesian citizens and 18–70 years of age. Twenty subjects were allocated as the case group (subjects with T2DM) and 20 subjects were allocated as the control group (subjects with normal glucose tolerance [NGT]). Both fasting intact GLP-1 (FGLP-1) and 60 minutes post-75 gram glucose loading intact GLP-1 (1hGLP-1) levels were measured.

**Results:**

Both fasting and post-prandial GLP-1 levels were significantly lower in subjects with T2DM than those with NGT (2.06 ± 0.43 *vs*. 2.87 ± 0.67 pg/L, p < 0.01; and 2.49 ± 0.60 *vs*. 3.42 ± 0.85 pg/L, p = 0.02; respectively). Low levels of FGLP-1 (OR, 13.5; p = 0.001) and 1hGLP-1 (OR, 5.667, p = 0.018), with no response after glucose loading (∆GLP-1), were a significant risk for T2DM. According to the ∆GLP-1, there was a tendency of decreasing response of GLP-1 after glucose loading among subjects with T2DM (∆ = 0.43 pg/L) compared to subjects with NGT (∆ = 0.55 pg/L).

**Conclusion:**

From this study it can be concluded that levels of intact GLP-1 are an important risk factor for T2DM in the Indonesian population.

## Background

Type 2 diabetes mellitus (T2DM) is a metabolic disease. It is a result of a combination of genetic predisposition, lifestyle and environment that are considered to be involved in its pathogenesis. Recently it has been widely known that changes of behavior, ways of life and the environment are predominant risk factors for T2DM [1]. The prevalence of T2DM in young adults is around 2.8 percent in 2000 and it is projected that the number of patients will increase from 171 million people in 2000 to approximately 366 million in 2030 [2]. In Bali, the prevalence of diabetes in adults was at 5.9% as reported by Suastika et al. in 2011 [3].

The concept that gut endocrine stimulates pancreatic secretion was first hypothesized in 1902. It was considered a big leap in the understanding of incretins that function as a regulator of blood glucose through regulation of insulin and glucagon secretion [4]. Two gut hormones, glucagon-like peptide-1 (GLP-1) and glucose dependent insulinotropic polypeptide or gastric inhibitory polypeptide (GIP), have been shown to act as incretins. GLP-1 has been developed as the basis of therapy for patients with T2DM. It was reported that in Caucasian patients with T2DM the GLP-1 levels decreased compared to that in normal subjects [5]. The total GLP-1 levels in among non-diabetic Japanese and Caucasians turned out to be comparable, but the intact GLP-1 levels was much lower in Japanese compared to Caucasians [6].

In Indonesia, no data on GLP-1 levels are available both in normal subjects and subjects with T2DM. Thus, this study was conducted to assess the GLP-1 levels in fasting and after glucose loading states and the response of GLP-1 after glucose loading; and to prove that low GLP-1 levels was a risk factor of T2DM in the Indonesian population.

## Methods

A case–control study was conducted at the Diabetes Clinic Sanglah Hospital, Denpasar, Bali, Indonesia, during the period of February to April 2014. Twenty cases (subjects with T2DM) and 20 subjects with normal glucose tolerance (NGT), aged 18–70 years, were enrolled in the study as the control group. All subjects were native Indonesian citizens. The diagnosis of diabetes was confirmed based on the criteria of ADA (2014) [7]. The levels of intact GLP-1 were measured in fasting (FGLP-1, at least 8 hours) and 60 minutes post-loading standard 75 gram anhydrous glucose (1hGLP-1) states. The levels of plasma intact GLP-1 were measured by the EIA kit, Cat.No.: RSCYK 160R, Bio Vendor Research and Diagnostic Products. Low level of GLP-1 was confirmed if the level of FGLP-1 was <2.21 pg/L; 1hGLP-1 was <2.57 pg/L; and the response or difference of fasting and post-prandial (∆GLP-1) was <0.029 pg/L. The values were determined by calculation of “mean-1SD” for each value (fasting, post-prandial and ∆) in subject with NGT or control group. This study protocol was approved by the Research Ethics Committee Faculty of Medicine, Udayana University, Denpasar, before the study was conducted (registration no.790/UN.4.2/Litbang/2013). Written informed consent was obtained from all participants.

Statistical tests used to analyze the data in the study included a descriptive presentation, a Mann–Whitney test, cross-tab (**χ**2 test and prevalence risk or odds ratio [OR]), with significant value confirmed at *P* < 0.05.

## Results

The mean age of the subjects was 56.85 ± 5.71 years (case, 57.05 ± 5.77 years and control, 56.65 ± 5.79 years, respectively). Body mass index in subjects with T2DM was higher than subjects with NGT, 28.94 ± 1.96 and 24.79 ± 3.07, respectively (*P* < 0.01).The mean of FGLP-1, 1hGLP-1, and ∆GLP-1 are seen in Table 1. Both FGLP-1 and 1hGLP-1 levels were lower significantly in subjects with T2DM than those with NGT (2.06 ± 0.43 *vs*. 2.87 ± 0.67 pg/L, *P* < 0.01; and 2.49 ± 0.60 *vs*. 3.42 ± 0.85 pg/L, *P* = 0.02; respectively).

**Table 1:** **Levels of GLP-1 among subjects with T2DM and normoglycemia**

Variable	T2DM (n = 20)	NGT (n = 20)	***P***
Fasting Plasma Sugar (mg/dl)	194.70 ± 10.50	82.60 ± 5.42	<0.01
Prandial Plasma Sugar (mg/dl)	315.35 ± 26.25	133.90 ± 8.55	<0.01
FGLP-1 (pg/L)	2.06 ± 0.43	2.87 ± 0.67	<0.01
1hGLP-1 (pg/L)	2.49 ± 0.60	3.42 ± 0.85	0.002
∆GLP-1 (pg/L)	0.43 ± 0.36	0.55 ± 0.52	0.715

T2DM, type 2 diabetes mellitus; NGT, normal glucose tolerance; FGLP-1, fasting GLP-1; 1hGLP-1, 1 hour after glucose loading GLP-1; ∆GLP-1, responses of GLP-1 after loading.

**Figure 1:**
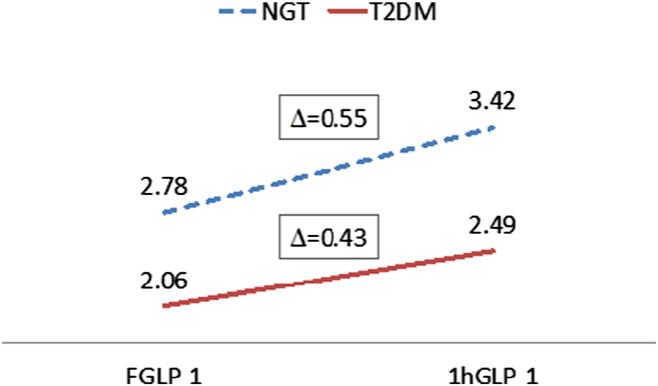
**Response of GLP-1 after 75 g glucose-loading T2DM, type 2 diabetes mellitus; NGT, normal glucose tolerance; FGLP-1, fasting GLP-1; 1hGLP-1, 1 h after glucose loading GLP-1.**

Low levels of FGLP-1 and 1hGLP-1 were more frequently found in subjects with T2DM than in subjects with NGT (60% *vs*. 10%, *P* = 0.001 and 50% *vs*. 15%, *P* = 0.018, respectively). Low levels of FGLP-1 (OR, 13.5; *P* = 0.001) and 1hGLP-1 (OR, 5.667, *P* = 0.018) were significantly a risk factor for T2DM, but not for ∆GLP-1 (Table 2). Although it has not been proven that ∆GLP-1 is a risk factor for T2DM, by looking at the response of GLP-1 after glucose loading, there seemed to be a tendency of decreasing response of GLP-1 among subjects with T2DM (∆ = 0.43 pg/L) compared to subjects with NGT (∆ = 0.55 pg/L) (Figure 1).

**Table 2:** **Risk of low FGLP-1, 1hGLP-1, and ∆GLP-1 levels for T2DM**

	T2DM	NGT	OR (95% CI)	***P***
*Fasting state*				
Low FGLP-1 (<2.21 pg/L)	12 (60%)	2 (10%)	13,5 (2.434-74.867)	0.001
Normal GLP-1 (≥2.21 pg/L)	8 (40%)	18 (90%)		
*Post-glucose loading state*				
Low 1hGLP-1 (<2.57 pg/L)	10 (50%)	3 (15%)	5.667 (1.254-25.606)	0.018
Normal 1hGLP-1 (≥2.57 pg/L)	10 (50%)	17 (85%)		
*Difference (*∆*)*				
Low ∆GLP-1 (<0.029 pg/L)	2 (10%)	2 (10%)	1.00 (0.127-7.893)	1.0
Normal ∆GLP-1 (≥0.029 pg/L)	18 (90%)	18 (90%)		

T2DM, type 2 diabetes mellitus; NGT, normal glucose tolerance; FGLP-1, fasting GLP-1; 1hGLP-1, 1 h after glucose loading GLP-1; DGLP-1, responses of GLP-1 after loading.

## Discussion

This study revealed that the levels of GLP-1 in fasting and post-prandial states in subjects with T2DM were lower than in subjects with NGT (2.06 *vs*. 2.87 pg/L and 2.49 *vs*. 3.42 pg/L, respectively). Velasquez-Mieyer et al. in 2008 reported that African-Americans exhibited lower GLP-1_active_ levels at 15 min after OGTT than Caucasians (3.75 ± 0.63 *vs.* 9.5 ± 4.5 pmol/L, *P* = 0.03) [8]. Levels of fasting GLP-1_active_ in African-Americans was lower than in Caucasians (3.31 ± 1.14 vs. 6.67 ± 3.84 pmol/L) but the difference was not statistically significant. As summarized by Seino et al. in 2010, the intact GLP-1 level was lower in Japanese than Caucasians [6]. Genetic or race background might influence the levels of GLP-1 since the levels widely vary among different races. In Japanese, fasting levels of total and intact GLP-1 were lower in patients with T2DM than the control group (15.5 ± 1.7 vs. 15.7 ± 1.0; and 0.2 ± 0.1 vs. 0.7 ± 0.2 pM, respectively) [9]. In Chinese adults the levels of total fasting GLP-1, 2hGLP-1 decreased significantly in prediabetes (impaired fasting glycemia [IFG] and impaired glucose tolerance [IGT]) and T2DM compared to normal glucose tolerance (NGT) and isolated IFG or IGT [10]. A similar finding showed Caucasian subjects with T2DM had decreased GLP-1 levels compared to the control subjects (23.8 ± 3.17 *vs.* 76.4 ± 4.47 pg/mL, *P* < 0.001) [11]. From the two review articles it can be summarized that low level of GLP-1 in T2DM is caused by impaired secretion of GLP-1 and accelerated metabolism of GLP-1. Low level or activity of GLP-1 in T2DM decrease an oral glucose dependent insulin secretion [12, 13].

Low levels of FGLP-1 (OR, 13.5) and 1hGLP-1 (OR, 5.667) seen in this study were significant risk factors for T2DM. Although the ∆GLP-1 was not a significant risk factor for T2DM, it revealed a trend of decreased response of intact GLP-1 after glucose loading among subjects with T2DM (0.43 pg/L) compared to subjects with NGT (0.55 pg/L). Two hours total GLP-1 increments (∆GLP-1) following OGTT reduced significantly in T2DM (4.26 ± 6.27 pmol/L) group compared to NGT (12.37 ± 10.05 pmol/L) and isolated IFG (10.37 ± 9.82 pmol/L) or IGT (9.79 ± 10.50 pmol/L) groups [10]. A similar result was also reported by Vilsboll et al. in 2003 that there was decreased GLP-1 responses after large meals in T2DM patients compared to healthy subjects [14].

Since there were differences in the type of GLP-1 (total or intact/active), method and unit GLP-1 measurement used in several different studies, there has been difficulty in comparing the absolute value between this study and other studies. However, it can be concluded that there was similarity in the findings that the levels of FGLP-1 and response after meal or glucose loading of GLP-1 were reduced in subjects with T2DM compared to subjects with NGT.

## Conclusion

Both FGLP-1 and 1hGLP-1 levels were lower in subjects with T2DM than in subjects with NGT. Low level of GLP-1 was an important risk factor of T2DM.

## Abbreviations

1hGLP-1: 1 hour after glucose loading GLP-1
ADA: American Diabetes Association
FGLP-1: Fasting GLP-1
GLP-1: Glucagon-Like Peptide-1
IFG: Impaired fasting glucose
IGT: Impaired glucose tolerance
NGT: Normal glucose tolerance
T2DM: Type 2 diabetes mellitus
OR: Odd ratio.
